# Taking integrated care forward: the need for shared values

**DOI:** 10.5334/ijic.1180

**Published:** 2013-06-24

**Authors:** Nick Goodwin

**Affiliations:** Editor-in-Chief, International Journal of Integrated Care

## Taking integrated care forward: the need for shared values

At our recent 13th International Conference on Integrated Care we examined a number of key challenges to the successful adoption of integrated care [1]. The focus of debate centred on four key systemic questions: what kinds of payment system best incentivise integrated care?; which organisational solutions are most effective?; how can care be better co-ordinated around people’s needs?; and what implementation strategies are likely to be most effective to stimulate change?

Underpinning the discussion was an acute sense that the adoption of integrated care principles into health and social care systems was too slow to meet the present and future needs of ageing populations with ever more complex and long-term medical problems. Keynote speakers variously described the need to adopt integrated care ‘at scale and pace’ [2] or ‘with speed and spread’ [3] and so outlined a range of potential strategies to support this. Hence, it was argued, short-term, small-scale, disease-based and organisationally-driven solutions should be avoided. Instead, strategies based on achieving long-term goals to the wider benefit of people and populations were needed backed up by political support, visionary leadership and the promotion of common values.

The issue of developing a collaborative culture has often been put forward as a key ingredient to making a success of integrated care. What was striking during the conference was the consistent emphasis placed on ‘creating energy for change’ through, for example: building ‘a community of peers to accelerate learning and improvement’ [3]; and ‘raise joy, professional pride and support, and recognise the real value [of their work] for society’ [4]. In other words, the ability to build social capital and promote engagement and learning between partners in care was argued to be a pre-requisite to accelerating the pace of adoption.

The importance of developing shared values in taking integrated care forward was driven home to me at a recent meeting to examine the collective experience and learning from five UK-based case studies of care co-ordination to people with complex needs [5]. A characteristic underpinning the success of each case study was the personal commitment of staff—both managers and professionals—to go that ‘extra mile’ by working beyond the boundaries of their job description in order to achieve the best results for their clients and in supporting colleagues to do the same. Lying behind this finding was a range of explicit strategies that promoted a strong ethos amongst staff to ‘do the right thing’—for example: promoting the needs of clients before themselves; supporting knowledge-sharing; and enabling role-substitution and subsidiarity through staff empowerment.

The problem with promoting the idea that a values-driven approach should be a pre-requisite to the successful adoption of integrated care is that the weight of both evidence and experience predicts that such a process requires considerable time and effort. Moreover, given the mismatch of motives that exists when integrating the work of professionals and organisations, such efforts often go unrewarded and/or require continual negotiation. Hence, rather than being perceived as a catalyst for change, leaders and managers tasked with applying integrated care ‘at scale and pace’ might instead focus on driving forward the organisational solution or introduce various financial inducements in the hope this will be more effective. Such an approach would be a mistake. When looking at successful implementation strategies in integrated care it is clear no short cuts exist—it takes visionary and stable leadership over the long-term to build the collaborative culture necessary to take integrated care forward.

As a final point, there was consensus during the conference that the ability to drive integrated care at a regional or local scale—and hence to make the necessary decision to invest the time and resources to build networks and alliances—relies on political will and commitment. Today, the principles of integrated care have become a high political priority for many national governments and regions. It is to be hoped that this political support will remain for the long-term since, as the history of integrated care tells us, successful adoption takes time as well as commitment.

Our 14th International Conference on Integrated Care, to be held in Brussels on 3–4 April 2014, will examine ‘taking integrated care forward’ as its major theme [6]. In particular, it will focus on how the right policies and change management strategies can support the ambition to accelerate the successful adoption of integrated care in practice.
